# Antibody persistence up to 5 y after vaccination with a quadrivalent meningococcal ACWY-tetanus toxoid conjugate vaccine in adolescents

**DOI:** 10.1080/21645515.2016.1248009

**Published:** 2017-02-02

**Authors:** Beatriz P. Quiambao, Ashish Bavdekar, Anand Prakash Dubey, Hemant Jain, Devayani Kolhe, Véronique Bianco, Jacqueline M. Miller, Marie Van der Wielen

**Affiliations:** a Clinical Research Division, Research Institute for Tropical Medicine, Alabang, Muntinlupa City, Philippines; b K.E.M Hospital, Sardar Moodliar Road, Pune, Maharashtra, India; c Department of Pediatrics, Maulana Azad Medical College (MAMC), and Associated Lok Nayak Hospital, New Delhi, India; d Chacha Nehru Hospital, Indore, India; e GSK Vaccines, Bangalore, India; Wavre, Belgium; and King of Prussia, PA, USA

**Keywords:** adolescents, antibody persistence, conjugate vaccine, *Neisseria meningitidis*, quadrivalent meningococcal vaccine

## Abstract

Long-term protection against meningococcal disease relies on antibody persistence after vaccination. We report antibody persistence up to 5 y after vaccination in adolescents who received a single dose of either meningococcal serogroups A, C, W, Y tetanus toxoid conjugate vaccine (MenACWY-TT, Pfizer) or MenACWY polysaccharide vaccine (MenPS, GSK Vaccines) at the age of 11–17 y in the randomized controlled primary study NCT00464815. In this phase III, open, controlled, multi-center persistence follow-up study conducted in India and the Philippines (NCT00974363), antibody persistence was evaluated by a serum bactericidal antibody assay using rabbit complement (rSBA) yearly, up to year 5 after vaccination. Serious adverse events (SAEs) related to study participation were recorded. Five years after a single dose of MenACWY-TT, the percentage of participants (N = 236) with rSBA titers ≥1:8 was 97.5% for serogroup A, 88.6% for serogroup C, 86.0% for serogroup W and 96.6% for serogroup Y. The percentages in the MenPS group (N = 86) were 93.0%, 87.1%, 34.9% and 66.3%, respectively. Exploratory analysis indicated a higher percentage of subjects with rSBA titers ≥1:8 for serogroups W and Y, and higher rSBA geometric mean antibody titers for serogroups A, W and Y in the MenACWY-TT group than the MenPS group at each time point (years 3, 4 and 5). No differences between groups were observed for serogroup C. No SAEs related to study participation were reported. In conclusion, the results of this follow-up study indicate that antibodies persisted up to 5 y after a single dose of MenACWY-TT in adolescents.

## Introduction

*Neisseria meningitidis* causes severe invasive disease, which typically presents as meningitis or septicemia.^1^ The incidence of invasive meningococcal disease (IMD) is the highest in infants and young children, but a secondary peak occurs during adolescence.^2,3^ Six serogroups (A, B, C, W, Y and X) are responsible for the majority of IMD worldwide, but their regional distribution varies and the predominant serogroup in any region can change over time.^4^ Since 1982, 7 countries in Asia (India, Indonesia, Mongolia, Nepal, Pakistan, the Philippines and Vietnam) have experienced IMD epidemics due to serogroups A or C, most recently in 2005 in the Philippines and India.^5-7^ Taiwan experienced a serogroup Y outbreak between 2001 and 2003, and serogroup W caused an outbreak among Hajj pilgrims and their contacts in Singapore in 2000–2001.^8,9^ While little is known about the epidemiology of sporadic IMD in Asian countries, the available data suggest that the burden may be substantial, particularly in developing countries in the region, and that serogroups C, Y and W are potentially increasing in importance.^4,7^

The burden of IMDs can be reduced through administration of effective meningococcal vaccines. Three quadrivalent meningococcal serogroups A, C, W and Y (MenACWY) conjugate vaccines are currently licensed for use. These vaccines differ in capsular polysaccharide content and carrier protein: *Nimenrix*™ (MenACWY-TT, Pfizer) contains 5 μg of each meningococcal capsular polysaccharide and is conjugated to tetanus toxoid; *Menactra*™ (MenACWY-DT, Sanofi Pasteur) contains 4 μg of each meningococcal capsular polysaccharide and is conjugated to diphtheria toxoid; and *Menveo*™ (MenACWY-CRM, GSK Vaccines) contains 10 μg of polysaccharide A and 5 μg each of the polysaccharides C, W and Y, and is conjugated to mutant diphtheria toxoid CRM_197_. For all 3 vaccines, immunization of adolescents is achieved using a single dose.

In the United States (US) where *Menactra*™ and *Menveo*™ are licensed for use, the Advisory Committee on Immunization Practices introduced a recommendation for administration of a booster dose of meningococcal conjugate vaccine 4–5 y after the initial vaccination during adolescence.^10^ This decision was based on preliminary US data showing that overall vaccine effectiveness among adolescents was estimated to decrease from 95% within the first year after vaccination to 58% between 2 and 5 y after vaccination.^10^ Available evidence suggests that immunity wanes over time regardless of the meningococcal conjugate vaccine used, with a decreasing percentage of adolescents who have seroprotective serum bactericidal antibody (SBA) titers up to 5 y after vaccination.^10-15^

In the primary vaccination study conducted in India, the Philippines and Taiwan (Clinicaltrials.gov NCT00464815),^16^ healthy adolescents 11–17 y of age who had not received any meningococcal vaccine within the previous 5 y were randomized (3:1) to receive a single dose of MenACWY-TT or a licensed quadrivalent meningococcal polysaccharide vaccine (MenPS; *Mencevax*™ACWY, Pfizer). One month post-vaccination, non-inferiority of MenACWY-TT compared to MenPS was demonstrated in terms of SBA titers using rabbit complement (rSBA). Vaccine response rates, and exploratory comparisons showed higher rSBA titers against all serogroups in MenACWY-TT recipients compared to MenPS recipients.

Since vaccine-specific antibody persistence data, along with estimates of long-term effectiveness, are needed to inform and guide the development of booster recommendations, all participants who completed the primary vaccination study in India and the Philippines were invited to return 2 y after vaccination for assessment of antibody persistence, and yearly thereafter until year 5 (NCT00974363). Persistence data at year 2 are presented elsewhere.^17^ Here, we report antibody persistence in this cohort of adolescents until 5 y after vaccination with either MenACWY-TT or MenPS.

## Results

**Figure 1.**
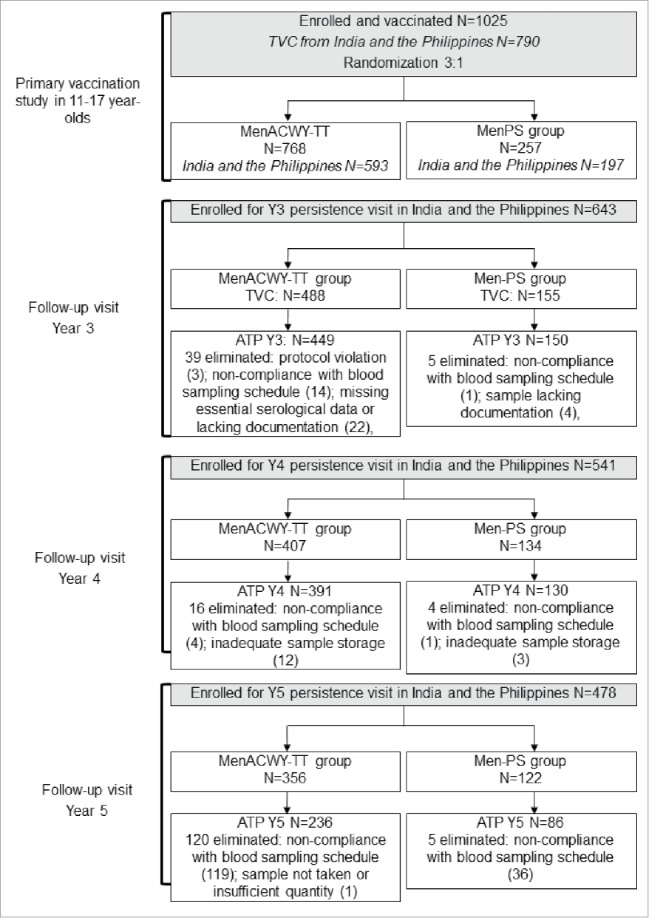
Flow of participants through the study. ATP = according-to-protocol cohort for persistence; TVC = total vaccinated cohort; N = number of participants in each group.

Of 790 eligible participants contacted from the primary study, 643 returned at year 3, 541 at year 4 and 478 at year 5 (Fig. 1). No participants were excluded because they had previously developed meningococcal disease. The median time since the primary vaccination was 61 months at year 5 (Table 1). Demographic characteristics of participants in the MenACWY-TT and MenPS groups were similar at year 3 and year 4 (Table 1). At year 5, 50.8% of participants in the MenACWY-TT group and 40.7% in the MenPS group were female.

**Table 1.** Summary of demographic characteristics at follow-up time points (according-to-protocol cohorts for persistence at year 3, year 4 and year 5).

		Year 3		Year 4		Year 5	
Characteristic		MenACWY-TT N = 449	MenPS N = 150	MenACWY-TT N = 391	MenPS N = 130	MenACWY-TT N = 236	MenPS N = 86
Age (years)	Mean (SD)	17.3 (2.0)	17.3 (2.0)	18.0 (2.0)	18.1 (2.0)	19.6 (1.9)	19.5 (2.0)
	Range	14–21	14–21	14–22	14–21	16–23	16–23
Gender,	Female	242 (53.9)	78 (52.0)	205 (52.4)	65 (50.0)	120 (50.8)	35 (40.7)
n (%)	Male	207 (46.1)	72 (48.0)	186 (47.6)	65 (50.0)	116 (49.2)	51 (59.3)
Central / South Asian heritage	n (%)	158 (35.2)	53 (35.3)	99 (25.3)	33 (25.4)	93 (39.4)	34 (39.5)
South East / Asian heritage	n (%)	291 (64.8)	97 (64.7)	292 (74.7)	97 (74.6)	143 (60.6)	52 (60.5)
Months since vaccination	Median (range)	37 (34–37)	37 (34–37)	46 (46–49)	47 (46–49)	61 (58–61)	61 (58–61)

N = total number of participants.

n/% = number / percentage of participants in a given category.

SD = standard deviation.

Five years after primary vaccination, the percentage of participants (N = 236) in the MenACWY-TT group with rSBA antibody titers ≥1:8 was 97.5% for serogroup A, 88.6% for serogroup C, 86.0% for serogroup W and 96.6% for serogroup Y (Table 2). In the group that received MenPS (N = 86), the percentages were 93.0%, 87.1%, 34.9% and 66.3%, respectively. At year 5, the exploratory analyses did not suggest any difference between groups in terms of the percentage of participants reaching these rSBA cut-offs for serogroup C, while for serogroup A, a higher percentage of participants reached the 1:128 cut-off in the MenACWY-TT group (Table 2). For serogroups W and Y, exploratory analysis suggested that a higher percentage of participants reached the rSBA cut-offs of 1:8 and 1:128 in the MenACWY-TT group than the MenPS group at each time point.

**Table 2.** Percentage of participants with rSBA titers ≥1:8 and ≥1:128 up to 5 y after vaccination with MenACWY-TT or MenPS at 11-–17 y of age (according-to-protocol cohorts for persistence at year 3, year 4 and year 5).

		MenACWY-TT					MenPS					Difference in % (MenACWY-TT minus MenPS)	
Serogroup	Time point	N	n	% ≥1:8(95% CI)	n	% ≥1:128(95% CI)	N	n	% ≥1:8 %(95% CI)	n	% ≥1:128(95% CI)	≥ 1:8 (95% CI)	≥ 1:128 (95% CI)
A	Y3	449	417	92.9 (90.1; 95.1)	398	88.6 (85.3; 91.4)	150	124	82.7 (75.6; 88.4)	118	78.7 (71.2; 84.9)	10.2 (4.3; 17.4)	10.0 (3.3; 17.7)
	Y4	391	353	90.3 (86.9; 93.0)	335	85.7 (81.8; 89.0)	130	105	80.8 (72.9; 87.2)	99	76.2 (67.9; 83.2)	9.5 (2.8; 17.6)	9.5 (2.0; 18.2)
	Y5	236	230	97.5 (94.5; 99.1)	219	92.8 (88.7; 95.7)	86	80	93.0 (85.4; 97.4)	71	82.6 (72.9; 89.9)	4.4 (−0.2; 12.0)	10.2 (2.6; 20.1)
C	Y3	449	409	91.1 (88.1; 93.6)	380	84.6 (81.0; 87.8)	150	129	86.0 (79.4; 91.1)	117	78.0 (70.5; 84.3)	5.1 (−0.5; 12.0)	6.6 (−0.3; 14.6)
	Y4	390	367	94.1 (91.3; 96.2)	347	89.0 (85.4; 91.9)	130	113	86.9 (79.9; 92.2)	104	80.0 (72.1; 86.5)	7.2 (1.7; 14.4)	9.0 (2.1; 17.2)
	Y5	236	209	88.6 (83.8; 92.3)	188	79.7 (74.0; 84.6)	85	74	87.1 (78.0; 93.4)	68	80.0 (69.9; 87.9)	1.5 (−5.9; 11.0)	−0.3 (−9.5; 10.5)
W	Y3	449	368	82.0 (78.1; 85.4)	350	78.0 (73.8; 81.7)	150	45	30.0 (22.8; 38.0)	36	24.0 (17.4; 31.6)	52.0 (43.4; 59.6)	54.0 (45.6; 61.2)
	Y4	390	301	77.2 (72.7; 81.3)	284	72.8 (68.1; 77.2)	130	35	26.9 (19.5; 35.4)	25	19.2 (12.8; 27.1)	50.3 (41.0; 58.3)	53.6 (44.8; 61.0)
	Y5	236	203	86.0 (80.9; 90.2)	195	82.6 (77.2; 87.2)	86	30	34.9 (24.9; 45.9)	26	30.2 (20.8; 41.1)	51.1 (39.6; 61.4)	52.4 (40.9; 62.3)
Y	Y3	449	418	93.1 (90.3; 95.3)	401	89.3 (86.1; 92.0)	150	87	58.0 (49.7; 66.0)	77	51.3 (43.0; 59.6)	35.1 (27.1; 43.4)	38.0 (29.6; 46.4)
	Y4	389	348	89.5 (86.0; 92.3)	333	85.6 (81.7; 88.9)	130	63	48.5 (39.6; 57.4)	60	46.2 (37.4; 55.1)	41.0 (31.9; 49.9)	39.5 (30.1; 48.5)
	Y5	236	228	96.6 (93.4; 98.5)	225	95.3 (91.8; 97.7)	86	57	66.3 (55.3; 76.1)	56	65.1 (54.1; 75.1)	30.3 (20.8; 41.1)	30.2 (20.5; 41.1)

Y3/4/5 = 3/4/5 y after vaccination, N = number of participants with available results, n = number of participants with titer equal to or above specified value, 95% CI = 95% confidence interval, rSBA testing carried out at PHE, Manchester, UK.

At each time point, exploratory analyses indicated that the rSBA geometric mean antibody titers (GMTs) for serogroups A, W and Y were higher in the MenACWY-TT group than in the MenPS group (Table 3).

**Table 3.** rSBA antibody titers 3, 4 and 5 y after vaccination with MenACWY-TT or MenPS at 11-17 y of age (according-to-protocol cohorts for persistence at year 3, year 4 and year 5).

		MenACWY-TT	MenPS	GMT ratio (ACWY-TT/MenPS)		
Serogroup	Time point	N	GMT(95% CI)	N	GMT(95% CI)	Ratio(95% CI)
A	Y3	449	448.3 (381.4; 527.1)	150	206.0 (147.4; 288.1)	2.2 (1.6; 3.1)
	Y4	391	386.9 (321.2; 466.2)	130	174.4 (121.2; 250.8)	2.2 (1.5; 3.3)
	Y5	236	643.8 (530.7; 781.0)	86	296.0 (202.4; 432.9)	2.2 (1.5; 3.1)
C	Y3	449	371.4 (309.4; 445.8)	150	389.8 (262.0; 579.9)	1.0 (0.7; 1.4)
	Y4	390	378.5 (319.7; 448.1)	130	364.0 (242.7; 545.9)	1.0 (0.7; 1.5)
	Y5	236	248.6 (194.2; 318.2)	85	366.5 (224.1; 599.4)	0.7 (0.4; 1.1)
W	Y3	449	338.0 (268.4; 425.6)	150	16.0 (10.9; 23.6)	21.1 (13.4; 33.3)
	Y4	390	209.8 (163.9; 268.6)	130	11.7 (8.2; 16.8)	17.9 (11.1; 28.7)
	Y5	236	436.9 (324.4; 588.4)	86	19.7 (11.8; 32.9)	22.2 (12.4; 39.5)
Y	Y3	449	740.5 (620.0; 884.3)	150	69.6 (44.6; 108.6)	10.7 (7.1; 15.9)
	Y4	389	533.4 (430.0; 661.7)	130	49.8 (30.7; 80.9)	10.7 (6.7; 17.0)
	Y5	236	1000.2 (824.1; 1214.0)	86	124.9 (71.2; 219.3)	8.0 (5.0; 12.6)

Y3/4/5 = 3/4/5 y after vaccination, N = number of participants with available results, 95% CI = 95% confidence interval, GMT = geometric mean antibody titer, rSBA testing carried out at PHE in Manchester, UK.

An increase in GMT was observed between year 4 and year 5 for serogroups A, W and Y. At year 5, the rSBA GMT for serogroup C remained similar, or lower, than that observed at year 4 (Table 3).

**Figure 2.**
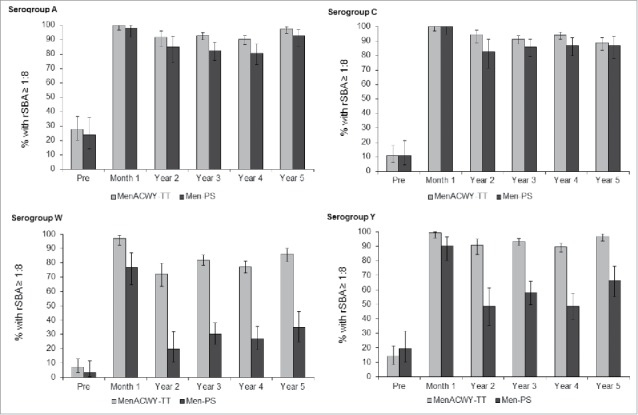
Percentage of participants with rSBA titers ≥1:8 over time. Footnote: *Post-hoc* analysis of a subset of samples from the according-to-protocol (ATP) cohort for immunogenicity (primary study) and the ATP cohort for persistence at year 2, all participants in the ATP cohorts for persistence at years 3, 4 and 5. Error bars show 95% confidence intervals. Pre = pre-vaccination; Month 1 = 1 month after vaccination; Year 2–5 = 2 to 5 y after vaccination.

**Figure 3.**
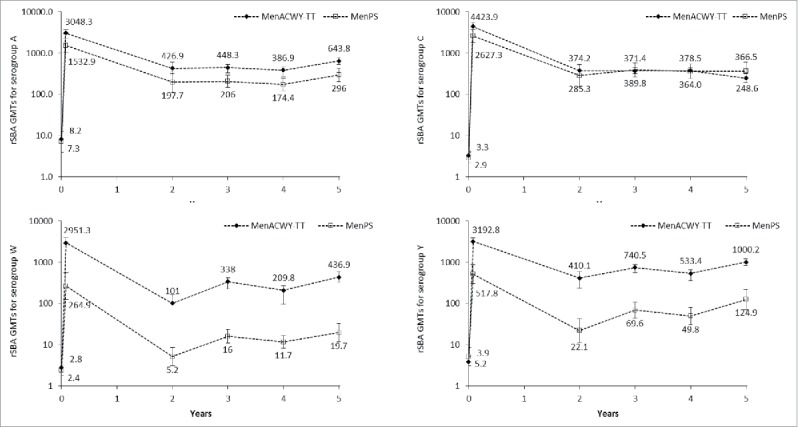
rSBA geometric mean titers (GMTs) over time. Footnote: *Post-hoc* analysis of a subset of samples from the according-to-protocol cohort (ATP) for immunogenicity (primary study) and the ATP cohort for persistence at year 2, all participants in the ATP cohorts for persistence at years 3, 4 and 5. Error bars show 95% confidence intervals. Time points shown are pre-vaccination, 1 month post-vaccination and 2 to 5 y after vaccination.

From year 3, the rSBA assay used to test the persistence of antibodies was performed at a different laboratory (Public Health England [PHE], Manchester, United Kingdom) than the rSBA assay used prior to year 3 (GSK laboratories). To allow a direct longitudinal comparison of antibody persistence in the 2 vaccine groups, a subset of samples (N = 756) collected prior to year 3 were re-tested at the PHE laboratory. The *post-hoc* analysis showed a sharper decline both in the percentage of participants with rSBA titers ≥1:8 (Fig. 2) and GMT values (Fig. 3), in the MenPS group compared to the MenACWY-TT group for serogroups W and Y. For each meningococcal serogroup, an increasing trend was observed in the percentage of subjects with rSBA titers ≥1:8 from the MenACWY-TT group, at each time point, starting from year 2. For both vaccines, rSBA GMTs for serogroups A and C persisted at similar levels between year 2 and year 5, with a small increase between year 4 and year 5 for serogroup A, while an increasing trend in rSBA GMTs was observed for serogroups W and Y (Fig. 3). No serious adverse events (SAEs) related to study participation were reported from the last visit of the primary vaccination study up to year 5.

## Discussion

This study evaluated antibody persistence in a large cohort of adolescents vaccinated up to 5 y previously with a single dose of quadrivalent MenACWY-TT. Antibody persistence appeared sustained, with at least 77.2% of vaccinees maintaining rSBA titers ≥1:8 for each serogroup at year 4, and at least 86.0% at year 5. In the ACWY-TT group, GMTs values observed for serogroup A, appeared higher compared to the MenPS group, while for serogroups W and Y, both GMTs and percentage of participants with rSBA titers ≥1:8 showed an ascending trend. At year 5, differences were maintained for serogroups W and Y in terms of percentage of subjects with rSBA titers ≥1:8 (86% and 96.6%) and A, W and Y in terms of GMTs (643.8 for serogroup A, 436.9 for serogroup W and 1000.2 for serogroup Y).

A small increase in rSBA GMTs between year 4 and year 5 was observed in both study groups for serogroups A, W and Y. Increases in rSBA over time for some serogroups after vaccination have been observed in other studies of MenACWY-TT conducted in other countries (Finland, Germany, US), and in different age groups.^15,18,19^ This may be suggestive of the development of natural immunity either through exposure to meningococci (nasopharyngeal carriage) or to bacteria that induce a cross-reactive response in the rSBA assay. However, meningococcal nasopharyngeal carriage is low in India,^20^ and serogroups W and Y are not commonly isolated from patients with IMD in India and South East Asia,^21^ suggesting that boosting by circulating meningococci may not have had a major impact on the observed rSBA titers in this study. The observed year-by-year oscillations in rSBA titers may also be explained by the assay variability and by the fact that subsequent visits were not always performed by the same participants.

Our results are consistent with previous studies of antibody persistence following primary vaccination with MenACWY-TT in adolescents and adults, in which persisting antibody levels after MenACWY-TT were similar or higher than after vaccination with MenPS.^18,19,22^ In industrialized countries, adolescence is associated with increased exposure to meningococci due to environmental and social behaviors favoring close contacts, such as dormitory living or night club attendance.^23,24^ IMD often occurs soon after nasopharyngeal acquisition of a new strain,^25,26^ although longer periods of asymptomatic carriage of meningococci have been reported.^27^ The incubation period of IMD is short, indicating that immune memory responses, which usually take at least 5 and up to 7 d to develop,^28,29^ may not occur soon enough to prevent fulminant invasive disease. In view of demonstrated vaccine failure in participants subsequently shown to have robust immune memory responses,^30^ maintenance of circulating antibodies is considered critical in providing ongoing protection against IMD.

Long-term antibody persistence has also been shown following vaccination with MenACWY-DT,^11,12^ and MenACWY-CRM.^11,13,31^ Since these studies were conducted in different laboratories and using different sources of complement, their results should be compared with caution, as only relative trends can be observed. In a head-to-head study assessing antibody persistence following primary vaccination with MenACWY-TT or MenACWY-DT vaccines,^31^ exploratory analyses suggested that the percentage of participants with SBA using human complement (hSBA) titers ≥1:8 at year 5 was higher for serogroup C in the MenACWY-TT-primed group than in the MenACWY-DT-primed group.^15^ Together, a growing body of data suggests that MenACWY-TT induces long-lasting immunity after a single dose in adolescents, that is at least as good, or better, than that induced by the MenACWY-DT vaccine.

Limitations of this study include the change in rSBA assay that occurred at year 3, which did not allow us to make direct comparisons with results obtained at earlier time points. However, we attempted to account for this by performing a *post-hoc* analysis on a subset of samples representative of the distribution of observed titers at each time point. This analysis supported the original conclusions of the primary vaccination study which was done using a rSBA assay at a different laboratory. This study was also limited by the fact that rSBA titers may vary over time during persistence studies. This observation may be due to the variability in the study population and to the fact that there was not sufficient serum to test a given persistence year time point with all previous time points. The variability in rSBA titers may also be due to boosting through asymptomatic carriage. Limitations of this study included also the long study duration that could have led to bias in results due to loss to follow-up and differences in relative retention between groups. However, the impact of this potential bias was probably limited since our modeling analysis, which included rSBA results for both groups, showed results in line with the observed rSBA antibody titers. Another limitation of this study is the fact that immune responses were only evaluated by an rSBA assay, while both rSBA and hSBA titers have been considered as correlates of efficacy for the licensure of meningococcal conjugate vaccines, and a correlation between rSBA and hSBA assays against serogroup C has been observed, it was not observed for the other serogroups.^32^ Higher titers are usually obtained with the rSBA assay because *N. meningitidis* are more easily lysed by rabbit complement.^33^ Finally, we were unable to compare immune responses with a control group that received another MenACWY conjugate vaccine because no such vaccine was licensed for use in the participating countries at the time of the study.

In conclusion, the 5-year persistence data indicate good long-term antibody persistence induced by a single dose of MenACWY-TT in adolescents.

## Methods

### Study details

The follow-up study from year 3 to year 5 took place between 28 August 2010 and 08 April 2013 (ClinicalTrials.gov NCT00974363). The primary study was conducted in the Philippines, India and Taiwan.^16^ Based on the enrolment for the primary study, participation from the Indian and Philippines sites was considered sufficient in terms of the sample size to be followed for antibody persistence. The two study centers in Taiwan were therefore not included in the follow-up study. There were 4 participating centers in India and one center in the Philippines at year 3, 3 participating centers in India and the Philippines site at year 4, and 2 centers in India and one in the Philippines at year 5. Participants were able to take part in any antibody persistence study visit 1, 2, 3, 4, or 5 independently of the other study visits, at their own or at their parent or legal guardian's discretion. They were not allowed to participate if they had developed meningococcal disease or received any meningococcal vaccination since the primary vaccination study.

The study protocols and associated documents were reviewed and approved by ethics committees in each participating country. The study was to be conducted in accordance with Good Clinical Practice (GCP), all applicable regulatory requirements and the Declaration of Helsinki. Each subject was to complete an assent or consent form, and their parent(s)/guardian(s) were to provide written informed consent if the subject was <18 years of age at the time of each visit.

During the conduct of the study, GSK Vaccines became aware of deviations from GCP at the study sites in India and the Philippines. These deviations and the actions taken by GSK are reported in Quiambao *et al*., 2016.^17^ At the year 3 and year 4 persistence time points the identified issues included improper consent and/or assent for 3 participants, and documentation lacking at one site in India confirming the appropriate storage of serum samples. From a subject rights perspective, the immunogenicity data of participants without proper informed consent and/or assent should not be used. Therefore, GSK performed a re-analysis of the data excluding all participants with improper consent. In addition, 27 participants at the year 3 and 15 participants at the year 4 time points were eliminated from the according-to-protocol (ATP) persistence cohort since proper storage of the samples could not be confirmed at this site, which could have affected immunogenicity results. The composition of the ATP cohorts at each time point is provided in Fig. 1.

### Immunogenicity assessment

Blood samples were collected from all participants who returned at each follow-up time point. Samples from year 3 onward were tested using a rSBA assay performed at the meningococcal surveillance laboratory of Dr. Ray Borrow at PHE in the (UK) and based on the Centers for Disease Control's protocol.^34^ The cut-off of the assay was a 1:8 dilution. An antibody titer ≥1:8 is considered indicative of seroprotection for meningococcal serogroup C,^35^ and was also applied to the other serogroups.^36^ A more conservative cut-off of 1:128 was also reported.

### Statistical analyses

The primary study objective at each time point was to evaluate the persistence of bactericidal antibodies up to 5 y after vaccination in terms of the percentage of participants in each group with rSBA titers ≥1:8 for each vaccine meningococcal serogroup. Secondary objectives included assessment of participants with persisting rSBA titers ≥1:128 and rSBA GMTs. SAEs considered by the investigator to be related to study participation or a GSK medication were captured retrospectively at each follow-up visit.

The primary analysis of persistence was done on the ATP persistence cohorts which included participants who had complied with all protocol-defined procedures and who had data available for at least one immunogenicity endpoint. Participants who were immunocompromised or who had received immunosuppressants or blood products including immunoglobulin were excluded from the ATP analyses.

A subset of samples obtained in the primary vaccination study (NCT00464815) and in the follow-up study at year 2 (NCT00955682) were re-tested at PHE. The samples were selected using a Gibbs sampling from the ATP immunogenicity and persistence cohorts, allowing a representative sample of the distribution of titers for each meningococcal antigen. One month after vaccination, participants from the ATP cohort for immunogenicity were ranked according to their results with the rSBA assay and classified in 25 consecutive classes, each representing 4% of the results. The participants were sampled per study group, per assay, and per time point. For each timepoint, the first 6 participants of the MenACWY-TT group and the first 3 participants of the MenPS group who had a sufficient quantity of serum were selected to be tested at PHE. The same subject was not necessarily sampled at each time point, given limitations in the volume of serum per subject for each time point.

For each vaccine serogroup, an exploratory evaluation of the differences in the immune response at each time point was performed between the MenACWY-TT and MenPS groups. In terms of differences in percentages of participants with rSBA antibody titers ≥1:8 and ≥1:128, 2 vaccine groups were considered potentially different if the standardized asymptotic 95% confidence intervals (CIs) for the differences in rates between the 2 vaccine groups did not contain the value “0.” In terms of GMTs, 2 vaccine groups were considered potentially different if the 95% CIs for the GMTs ratios between the 2 vaccine groups, which were computed using an Analysis of Variance (ANOVA) model on the log_10_-transformed titers with the vaccine group and country as fixed effects in the model (pooled variance), did not contain the value “1.” Note that potential differences should be interpreted with caution considering that there was no adjustment for multiplicity for these comparisons, and that significant findings may have occurred by chance alone.

Statistical analyses were performed using SAS software (SAS Institute Inc., Cary, NC, US) version 9.22.

### Trademarks

MENVEO is a registered trademark of the GSK group of companies. MENACTRA is a registered trademark of Sanofi Pasteur. MENCEVAX is a registered trademark of Pfizer. NIMENRIX is a registered trademark of the GSK group of companies, licensed to Pfizer.

## Abbreviations

ATP: According–to-protocol
CI: Confidence interval
CRM: cross-reactive material
DT: Diphtheria toxoid
GMT: Geometric mean antibody titer
IMD: Invasive meningococcal disease
rSBA: Serum bactericidal antibody assay using rabbit complement
MenACWY-TT: Pfizer's quadrivalent meningococcal serogroups A, C, W and Y vaccine conjugated to tetanus toxoid
MenPS: Quadrivalent meningococcal serogroups A, C, W and Y polysaccharide vaccine
PHE: Public Health England
SD: Standard deviation
TT: Tetanus toxoid
UK: United Kingdom
US: United States
